# Alzheimer’s Disease: Because Amyloid‐β Cannot Differentiate Between Bacteria and Neurons

**DOI:** 10.1002/alz.088345

**Published:** 2025-01-03

**Authors:** Matthew P Neal, Autumn Meek, Donald F Weaver

**Affiliations:** ^1^ Krembil Research Institute, Toronto, ON Canada

## Abstract

**Background:**

An explicit molecular level understanding of Alzheimer’s Disease (AD) remains elusive. What initiates the disease and why does it progress? Answering these questions will be crucial to the development of much needed new diagnostics and therapeutics. Though the amyloid hypothesis is often debated, recent biologic trial results support a role for Aβ in AD pathogenesis. However, there are other tenable hypotheses, most notably neuroinflammation, but also gliopathy, synaptopathy, mitochondriopathy, and oxidative stress. Is it possible to formulate a model of AD that incorporates multiple proposed hypotheses into a single unifying conceptual approach? We seek to answer this question.

**Method:**

We performed a comprehensive series of *in silico*, *in vitro* and *in vivo* studies explicitly evaluating the atomistic‐molecular mechanisms of Aβ‐mediated neurotoxicities as well as Aβ’s antimicrobial and immunomodulatory effects. The molecular effects of Aβ and various cytokines on mitochondria, neuronal membranes and synapses were also explicitly studied.

**Result:**

Membranes are a mosaic of lipophilic and hydrophilic (negatively‐charged) regions organized in specific geometric patterns. Our studies show that bacterial membranes and neuronal membranes have essentially identical patterns, making neurons inadvertently susceptible to molecules targeting bacterial membranes. From this, a new model of AD emerges: In response to various immunostimulatory events (infection, trauma, ischemia, diabetes, air pollution), Aβ is released as an immunopeptide (kinocidin‐type cytokine) which exhibits both immunomodulatory and antimicrobial properties (whether bacteria are present, or not); this inflicts a misdirected attack upon ‘self’ neurons, arising from the essentially identical membrane surface electrotopologies between neurons (especially at the synapse) and bacteria – causing neuronal death by mistaken identity. Following this self‐attack, the resulting necrotic neuronal breakdown products diffuse to adjacent neurons eliciting further release of Aβ, leading to a self‐perpetuating cycle.

**Conclusion:**

We propose that AD occurs because Aβ is an immunopeptide that cannot differentiate neurons from bacteria – a case of mistaken identity that leads to an innate autoimmune response in which Aβ extracellularly attacks neuronal membranes, particularly at the synapse, while also intracellularly attacking mitochondria.

